# Levofloxacin-associated hypoglycaemia complicated by pontine myelinolysis and quadriplegia

**DOI:** 10.1111/j.1464-5491.2008.02465.x

**Published:** 2008-07

**Authors:** S Vallurupalli, G Huesmann, J Gregory, M G Jakoby

**Affiliations:** * Department of Internal Medicine, University of Illinois College of Medicine at Urbana-ChampaignIL; † Department of Surgery, University of Illinois College of Medicine at Urbana-ChampaignIL; ‡ Department of Internal Medicine, University of Nevada School of MedicineLas Vegas, NV, USA

**Keywords:** central pontine myelinolysis, fluoroquinolones, hypoglycaemia, quadriplegia, Type 2 diabetes mellitus

## Abstract

**Background:**

Central pontine myelinolysis (CPM) usually presents in chronic alcoholics and in patients in whom hyponatraemia has been corrected rapidly. However, CPM may occur in other clinical circumstances, including patients with severe hypoglycaemia. We describe the occurrence of CPM and quadriplegia in a patient who experienced fluoroquinolone-associated severe hypoglycaemia.

**Case report:**

A 63-year-old man with Type 2 diabetes mellitus was admitted to hospital for resection of a large liposarcoma. Renal-dose levofloxacin was utilized as part of an antimicrobial regimen to treat post-operative peritonitis. On days 6–8 of levofloxacin therapy, the patient experienced recurrent hypoglycaemia despite total parenteral nutrition, 10% dextrose containing fluids and cessation of insulin therapy 3 days prior to the first hypoglycaemic episode. Hypoglycaemia resolved within 24 h of stopping levofloxacin. After a final and severe hypoglycaemic event, the patient developed quadriplegia and tonic left deviation of gaze. Magnetic resonance imaging revealed a high-intensity lesion in the central pons consistent with CPM.

**Conclusions:**

Fluoroquinolones should be considered as a potential cause of hypoglycaemia. Severe hypoglycaemia has the potential to cause white matter lesions in the pons. Putative mechanisms include failure of membrane ion channels, oligodendrocyte apoptosis and oxidative stress of glucose reperfusion. Fluoroquinolone-associated hypoglycaemia and hypoglycaemia-induced quadriplegia are both rare and we believe this is the first case report linking the two events.

Diabet. Med. 25, 856–859 (2008)

## Introduction

Central pontine myelinolysis (CPM) is a serious neurological disorder of brain white matter initially described in alcoholics and malnourished patients [1]. It may also occur if hyponatraemia is corrected too rapidly [2]. Liver transplantation and haemodialysis patients have accounted for an increasing proportion of cases in recent years [3]. Although rare, hypoglycaemia has been reported to cause CPM [4–6]. The exact pathogenesis of the disorder remains unclear.

Fluoroquinolones are a class of antibiotics that inhibit bacterial DNA synthesis and are used to treat a wide variety of infections. Disorders of glucose homeostasis have been reported in association with fluoroquinolone therapy, in particular gatifloxacin and levofloxacin. A report of two population-based, nested case–control studies of elderly adults treated in hospital for hypoglycaemia or hyperglycaemia after ambulatory treatment with a fluoroquinolone, cephalosporin, or macrolide found significantly increased risks of both hypoglycaemia and hyperglycaemia for gatifloxacin-treated patients and an increased risk of hypoglycaemia for patients treated with levofloxacin [7]. Gatifloxacin was withdrawn from the US and Canadian markets in June 2006 because of treatment-related glucose disturbances.

We present the case of a patient who experienced recurrent hypoglycaemia while receiving levofloxacin. After a severe hypoglycaemic event, the patient developed quadriplegia as a result of CPM.

## Case report

A 63-year-old man presented to hospital with increasing abdominal distension. He had Type 2 diabetes mellitus, managed with glibenclamide. A large, low-grade liposarcoma was removed along with the involved left kidney. Tumour resection was complicated by bowel perforation and three additional surgical procedures were performed for partial bowel resection and peritoneal debridement. Fluconazole, metronidazole and renal-dosed levofloxacin were administered for treatment of peritonitis. Adrenal insufficiency was diagnosed and managed with stress-dose parenteral hydrocortisone (50 mg every 8 h). Renal function worsened after left nephrectomy (peak creatinine 361 µmol/l), but electrolytes remained unremarkable and no signs of uraemia or fluid overload were observed. During a prolonged stay in the Intensive Care Unit (ICU), total parenteral nutrition (TPN, 0.83 mol/l glucose, 104 ml/h) was started and an ICU insulin infusion protocol was utilized to manage hyperglycaemia. The patient gradually recovered, permitting transfer to an Intermediate Care Unit (IMCU) on hospital day 22. TPN was continued, but the insulin infusion was stopped after transfer to the IMCU, with the patient receiving only insulin aspart (Novolog) according to a supplemental subcutaneous insulin protocol.

**FIGURE 1:**
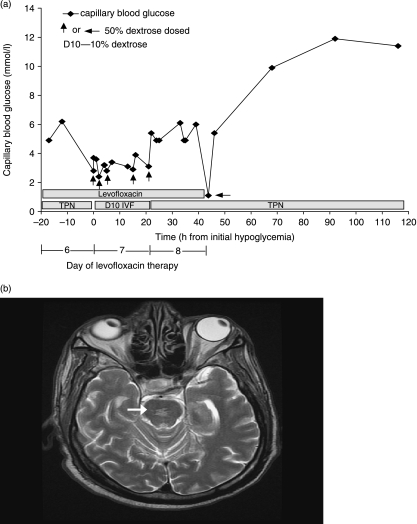
Time course of hypoglycaemia during levofloxacin therapy (a) and MRI imaging of the brain (b). (a) Recurrent episodes of hypoglycaemia occurred during days 6–8 of levofloxacin despite delivery of glucose in TPN or fluids. The last episode induced a brief cardiopulmonary arrest. (b) T2 weighted MRI image of the brain 2 days after the onset of quadriplegia. The lesion in the central pons (arrow) is consistent with central pontine myelinolysis. Note the deviation of the patient's eyes suggesting bulbar involvement. D10, 10% dextrose; IVF, intravenous fluids; MRI, magnetic resonance image; TPN, total parenteral nutrition.

On the 27th day of hospital stay, TPN was stopped because of a malfunctioning venous catheter. Two hours later the patient experienced neuroglycopenic symptoms and capillary blood glucose was 2.8 mmol/l. Symptoms abated after intravenous administration of 25 ml 50% dextrose. Low capillary blood glucose values were corroborated by a laboratory plasma glucose measurement. There was no insulin in the patient's TPN and the last dose of insulin aspart (4 units) had been administered subcutaneously 5 days before hypoglycaemia occurred. Ten per cent dextrose (D10) infusion at 104 ml/h was started after the initial episode of hypoglycaemia. Despite administration of D10 fluids, the patient required four additional doses of 50% dextrose to treat recurrent hypoglycaemia on hospital days 27 and 28 (days 6 and 7 of levofloxacin, see Fig. 1).

TPN was restarted during the evening of hospital day 28 and D10 fluids were stopped. On hospital day 29 (levofloxacin day 8), the patient suddenly developed cardiac asystole and required rapid intubation and cardiopulmonary resuscitation. Capillary blood glucose was 1.1 mmol/l at the time of cardiac arrest and multiple doses of 50% dextrose were administered to achieve euglycaemia. Sinus rhythm, normal blood pressure and spontaneous respiration were restored within 5 min of the event. However, 24 h later, the patient developed quadriplegia and tonic ocular deviation to the left. Magnetic resonance imaging (MRI) revealed a focal high intensity lesion on T-2 weighted images of the central pons (Fig. 1). The lesion also exhibited prominent intensity on diffusion-weighted images and reduced intensity on apparent diffusion coefficient (ADC) mapping. No cortical lesions were observed.

Because of concern about drug-induced hypoglycaemia, levofloxacin was discontinued. The patient required insulin therapy for hyperglycaemia within 24 h of stopping levofloxacin. No other aetiology of hypoglycaemia was identified. The patient's last dose of sulphonylurea was prior to admission, 27 days before the onset of hypoglycaemia in hospital and no insulin was administered in the 5 days before hypoglycaemia.

The patient gradually recovered partial motor function by the time of hospital discharge with the help of physiotherapy. Tracheotomy and gastrostomy were performed before transfer to a nursing home. Unfortunately, the patient returned to the hospital 1 month later in respiratory failure as a result of aspiration pneumonia and subsequently died.

## Discussion

To our knowledge, this is the first case of fluoroquinolone-associated hypoglycaemia complicated by pontine demyelination and quadriplegia. No alternative explanations are apparent and hypoglycaemia remitted quickly after levofloxacin was discontinued. In particular, the patient was not receiving exogenous insulin when hypoglycaemia occurred, dextrose was provided in either TPN or intravenous fluids during the episodes and hydrocortisone at stress doses was prescribed for adrenal insufficiency. Although high doses of fluconazole can increase circulating sulphonylurea levels and precipitate hypoglycaemia [19], our patient had not received any oral glucose-lowering medication for nearly 1 month before hypoglycaemia occurred. We are unaware of any reports documenting metronidazole-induced hypoglycaemia. The persistent and severe hypoglycaemia and lack of response to a D10 infusion point to a mechanism other than the discontinuation of TPN. There were no significant shifts in sodium or potassium to provide an alternative explanation for CPM. Hypoglycaemia is a potential cause of cardiac asystole and asystole resolved after euglycaemia was restored.

Patients most at risk of fluoroquinolone-induced hypoglycaemia are those with Type 2 diabetes mellitus treated with sulphonylureas [8,9]. Hypoglycaemia typically occurs 4–6 days after initiation of a fluoroquinolone antibiotic. Quinolones have been shown to directly stimulate insulin secretion from pancreatic B-cells, with at least part of this action occurring by direct action at pore forming KIR 6.2 subunits of K ATP channels [10]. In our patient's case, hypoglycaemia occurred on day 7 of levofloxacin therapy and resolved promptly after levofloxacin was stopped.

Severe hypoglycaemia may cause neurological disturbances ranging from focal deficits to coma and death. However, reports of hypoglycaemia-induced quadriplegia are rare in the English language literature [11–13]; one case of quadriplegia occurred as part of the ‘locked-in’ syndrome [11] and neurological symptoms in the other two cases resolved quickly after hypoglycaemia was treated [12,13]. None of these previous reports has documented pontine lesions on central nervous system (CNS) imaging.

Shirayama *et al*. have reported a case of intentional insulin-induced hypoglycaemia complicated by left hemiplegia and a pontine lesion with increased signal intensity on diffusion-weighted MRI [14]. Hypoglycaemia-induced CPM has also been reported in patients presenting in coma [4,5] and with ataxia [6]. Our patient's only acute lesion on CNS imaging was an area of high signal intensity in the pons characteristic of CPM. The patient's neurological findings can be explained by disruptions of the corticospinal tract, corticobulbar tract and paramedian pontine reticular formation.

There are three putative mechanisms by which severe hypoglycaemia could cause pontine myelinolysis. Deprivation of glucose, the major source of fuel for the brain, may lead to cytotoxic oedema secondary to failure of ion pumps in the cell membrane [15]. In animal models [15] and at least one patient [16], enhancement on diffusion-weighted MRI and decreased signal on ADC mapping as observed for our patient identified significant changes in brain water diffusion. Glucose resuscitation resulting in hyperglycaemia activates the NADPH pathway in neurons, causing cytotoxic oxidative stress [17]. The same phenomenon could also adversely affect oligodendrocytes. Finally, hypoglycaemia induces apoptosis pathways in oligodendrocytes and prevents the maturation of oligodendrocyte precursor cells [18].

We believe our case represents hypoglycaemia associated with fluoroquinolone therapy in a critically ill patient with renal failure. This case also illustrates two rare complications of hypoglycaemia, namely CPM and quadriplegia. CPM appears to be the aetiology of the patient's quadriplegia. More studies are required to elucidate the pathogenesis of white matter brain injury in hypoglycaemia.

## Abbreviations

ADC: apparent diffusion coefficient
CNS: central nervous system
CPM: central pontine myelinolysis
ICU: intensive care unit
IMCU: intermediate care unit
MRI: magnetic resonance imaging
TPN: total parenteral nutrition
